# Spiking neural networks for EEG signal analysis using wavelet transform

**DOI:** 10.3389/fnins.2025.1652274

**Published:** 2025-10-16

**Authors:** Li Yuan, Jian Wei, Ying Liu

**Affiliations:** Academy of Military Sciences, Beijing, China

**Keywords:** spiking neural networks, EEG signal analysis, brain-computer interfaces, discrete wavelet transform, bio-inspired methods

## Abstract

**Introduction:**

Brain-computer interfaces (BCIs) leverage EEG signal processing to enable human-machine communication and have broad application potential. However, existing deep learning-based BCI methods face two critical limitations that hinder their practical deployment: reliance on manual EEG feature extraction, which constrains their ability to adaptively capture complex neural patterns, and high energy consumption characteristics that make them unsuitable for resource-constrained portable BCI devices requiring edge deployment.

**Methods:**

To address these limitations, this work combines wavelet transform for automatic feature extraction with spiking neural networks for energy-efficient computation. Specifically, we present a novel spiking transformer that integrates a spiking self-attention mechanism with discrete wavelet transform, termed SpikeWavformer. SpikeWavformer enables automatic EEG signal time-frequency decomposition, eliminates manual feature extraction, and provides energy-efficient classification decision-making, thereby enhancing the model's cross-scene generalization while meeting the constraints of portable BCI applications.

**Results:**

Experimental results demonstrate the effectiveness and efficiency of SpikeWavformer in emotion recognition and auditory attention decoding tasks.

**Discussion:**

These findings indicate that SpikeWavformer can address the key limitations of existing BCI methods and holds promise for practical deployment in portable, resource-constrained scenarios.

## 1 Introduction

Brain-computer interfaces (BCIs) enable direct communication between the human brain and machines through electroencephalography (EEG) signal processing (Zhang et al., 2020). A typical BCI architecture comprises four functional modules: data acquisition, preprocessing, classification, and a feedback module (Lotte and Guan, 2010). BCI systems have demonstrated extensive real-world applicability in diverse domains including robotic manipulation (Liu et al., 2015), cognitive signal decoding (Cai et al., 2021), and neuropsychiatric interventions for emotional regulation (Zotev et al., 2020; Xing et al., 2019). As a common learning-based BCI method, deep learning methodology has demonstrated superior performance over conventional machine learning approaches across diverse BCI tasks (Ang et al., 2008; Wang et al., 2015), including motor imagery classification (Schirrmeister et al., 2017; Kwon et al., 2019), mental workload monitoring (Jiao et al., 2018), auditory attention decoding (Faghihi et al., 2022; Cai et al., 2024), and emotion recognition (Alarcao and Fonseca, 2017; Li et al., 2018). Nevertheless, previous research has predominantly relied on manually extracted EEG features such as power spectral density (PSD) and differential entropy (DE) (Jiao et al., 2018; Song et al., 2018; Zhong et al., 2020), whose limitations become increasingly evident. First, these feature extraction paradigms exhibit strong dependence on domain-specific knowledge (Singh and Krishnan, 2023; Subasi, 2019), necessitating task-specific extraction pipelines tailored to distinct experimental protocols, thereby compromising model generalizability across tasks. Second, manually crafted features often fail to capture nonlinear interrelationships in EEG time-frequency characteristics and multiscale dynamic properties (Singh and Krishnan, 2023; Vallabhaneni et al., 2021), potentially leading to critical information loss.

Wavelet Transform (WT) has emerged as a fundamental signal processing tool in EEG analysis (Grobbelaar et al., 2022) due to its unique time-frequency analysis capabilities. Unlike conventional Fourier Transform that provides only global frequency-domain information, WT enables multi-scale decomposition through its inherent multi-resolution analysis. This capability permits simultaneous signal characterization at distinct resolution levels-capturing macroscopic patterns (e.g., global trends) at coarse-grained scales while resolving microscopic fluctuations (e.g., localized variations) at fine-grained scales when processing electroencephalographic (EEG) signals. Furthermore, WT achieves adaptive hierarchical representation of non-stationary neural activities by dynamically adjusting the scale and translation parameters of basis functions, thereby effectively characterizing both transient features (e.g., high-frequency oscillations in event-related potentials) and long-range rhythmic patterns (e.g., sustained α-wave oscillations). Although recent years have witnessed preliminary applications of wavelet transform methodologies in EEG classification tasks. However, their predominant reliance on deep neural networks (DNNs) introduces computationally and resource-intensive demands, conflicting with the low-power objectives of resource-constrained portable BCI devices. Consequently, achieving optimal trade-offs between classification performance, system portability, and energy efficiency remains a critical challenge in practical BCI implementations.

**Figure 1:**
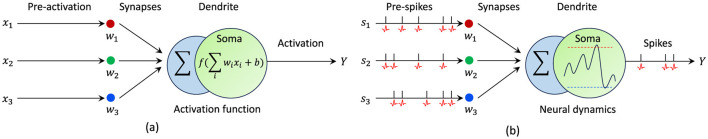
Comparison of neuron models in deep neural networks (DNNs) and spiking neural networks (SNNs). **(a)** Conventional DNNs neuron model processes continuous-valued inputs, where *x* represents input activations, *w* denotes synaptic weights, *b* is the bias term, and *Y* corresponds to the output activation. **(b)** Typical spiking neuron model that processes discrete spike events, with *s*_*i*_ representing input spikes, *w* indicating synaptic weights, and *Y* signifying the output spike train.

Spiking neural networks (SNNs), recognized as third-generation neural networks, have emerged as a promising alternative in BCI research due to their biologically plausible computation paradigm (Izhikevich, 2003; Maass, 1997; Masquelier et al., 2008). As shown in Figure 1, instead of continuous activations in deep neural networks (DNNs), SNNs employ discrete spike events as neuronal communication media, where spiking neurons activate exclusively upon reaching threshold potentials and remain quiescent otherwise (Gerstner and Kistler, 2002). This event-driven mechanism (Wei et al., 2024) facilitates synaptic computation sparsity while eliminating multiply-accumulate (MAC) operations, thereby achieving superior energy efficiency, which is critical for portable neurotechnological devices. Notably, SNNs have demonstrated remarkable success across multiple computational neuroscience domains in recent years. For instance, the energy-efficient Spike Transformer architectures proposed by Yao et al. (2023, 2024, 2025) and Zhou et al. (2022, 2023) have demonstrated exceptional performance in image classification (Deng et al., 2022; Shi et al., 2024), detection (Luo et al., 2024; Wang et al., 2025), and segmentation (Lei et al., 2025). Similarly, the SNN-based audio processing models developed by Wu et al. (2018); Pan et al. (2020); Wang et al. (2024) have made significant advancements in signal processing and keyword recognition. These successes establish a solid foundation for the broader adoption and cross-domain application of SNNs.

In this paper, we propose a novel BCI signal analysis framework that integrates wavelet transform with an spiking self-attention mechanism. This framework enables dynamic modeling and efficient computation of non-smooth EEG signals by combining brain-inspired spiking neural networks with the global-local feature extraction capabilities of the wavelet domain. Our approach not only overcomes the limitations of traditional manual feature extraction but also demonstrates, for the first time, the synergistic effectiveness of spiking self-attention and wavelet transform in cross-task scenarios through end-to-end training. In experimental evaluations focused on emotion recognition and auditory attention decoding tasks, our method achieves outstanding performance. The main contributions of this work are summarized as follows:

- We propose a novel spiking self-attention module integrated with discrete wavelet transform (DWT) for EEG signal processing. This innovative module simultaneously captures global rhythmic patterns and local transient features through multi-scale wavelet decomposition. Leveraging the spatio-temporal dynamics of spiking neurons, it effectively models nonlinear feature dependencies while replacing traditional Transformer's dense attention with efficient sparse pulse sequences.
- We present SpikeWavformer, the first end-to-end spiking neural network framework specifically designed for multi-task BCI analysis. The framework unifies time-frequency decomposition, dynamic feature selection, and classification within a biologically plausible computational paradigm. Its cascade architecture combines reversible wavelet transforms with spiking self-attention layers, enabling adaptive optimization across diverse BCI tasks including emotion recognition and auditory decoding.
- We conduct comprehensive evaluations on multiple public benchmark datasets to validate the effectiveness of SpikeWavformer. Experimental results demonstrate superior performance compared to existing methods, particularly in resource-constrained environments. The framework shows significant practical potential for real-world BCI applications, achieving state-of-the-art results while maintaining low computational overhead.

## 2 Related works

### 2.1 SNNs for EEG signal processing tasks

EEG-based BCIs have demonstrated significant potential across various downstream tasks, with auditory attention decoding (AAD) and emotion recognition representing two prominent application domains. In AAD research, the challenge stems from the cocktail party effect—the neurocognitive ability to selectively focus on target speakers in multi-talker environments (Cherry, 1953), which contrasts with difficulties experienced by hearing-impaired populations (Cai et al., 2024). Neurophysiological signal analyses through ECoG (Mesgarani and Chang, 2012), MEG (Akram et al., 2016), and EEG (O'sullivan et al., 2015) have enabled AAD implementations, catalyzing developments in neuro-steered hearing aids (Ceolini et al., 2020). For emotion recognition, the field seeks to model higher-order cognitive functions encoded in neurophysiological signals (Tan et al., 2021). While emotional states manifest through various modalities, the susceptibility of physical expressions to masking effects positions non-invasive EEG as a robust solution for emotion decoding (Xu et al., 2024; Li et al., 2019).

SNNs have emerged as a promising computational framework for both applications, leveraging their inherent low-latency processing and energy-efficient characteristics. In AAD research, Faghihi et al. (2022) developed efficient left/right attention pattern decoding, while Cai et al. (2023) proposed BSAnet, integrating biologically plausible mechanisms with attention modeling for temporal dynamics capture. Recent advances include spiking GCNs for spatial feature extraction (Cai et al., 2024), demonstrating promising results in low-density electrode scenarios. In emotion recognition, pioneering SNN applications have shown methodological viability. Tan et al. (2021) implemented NeuroSense achieving 78.97%/67.76% (arousal/valence) accuracy on DEAP, while Alzhrani et al. (2021) attained 94.83% accuracy using bidirectional spiking networks on DREAMER. Recent developments include fractal SNN architectures (Li et al., 2023), SGLNet for spatiotemporal extraction (Gong et al., 2023), and EESCN achieving 94.81% accuracy on DEAP and SEED-IV (Xu et al., 2024). However, previous research has predominantly relied on manually extracted EEG features such as power spectral density (PSD) and differential entropy (DE) (Jiao et al., 2018; Song et al., 2018), and automatic EEG feature extraction in this domain remains largely unexplored.

### 2.2 Spiking self attention mechanism

Traditional SNNs, despite their inherent advantages in energy efficiency and biological plausibility, still exhibit a performance gap compared to their DNN counterparts. Therefore, many recent works have integrated attention mechanisms into SNNs to enhance their performance and capabilities (Yao et al., 2021; Zhu et al., 2024; Zhou et al., 2024; Lu et al., 2025). Yao et al. (2023) addressed this through Spike-Driven Self-Attention (SDSA), reformulating matrix multiplications as masking operations to ensure purely binary spike signal transmission. Building on this foundation, Yao et al. (2024) introduced the Meta-Spikeformer architecture that extended the SDSA operator. Those advancement inspired subsequent research exploring SNN-specific attention mechanisms. Wang et al. (2023) proposed Spatiotemporal Self-Attention (STSA) for SNNs, maintaining asynchronous transmission while capturing spatiotemporal feature dependencies. More recently, Wang et al. (2025) developed Saccade Spike Self-Attention (SSSA), enabling comprehensive spatiotemporal feature processing for holistic visual scene understanding in SNN paradigms. Overall, these novel spiking self-attention mechanisms have significantly advanced SNN performance. However, there remains a lack of effective spiking self-attention designs specifically tailored for EEG signal processing.

## 3 Preliminary

### 3.1 Leaky integrate-and-fire neuron

SNNs rely on spiking neurons (Maass, 1997) as their basic unit of information transfer, and common spiking neurons include the Hodgkin-Huxley (Abbott and Kepler, 2005), Izhikevich (Izhikevich, 2003), and Leaky Integrate-and-Fire (LIF) (Izhikevich, 2003) model. In this work, we use the LIF model as the spiking neuron in the proposed method. The LIF model is a simple and effective spiking neuron model. When the membrane potential reaches a certain threshold, the neuron emits a spike, followed by a reset of the membrane potential to the resting potential *V*_*reset*_. The dynamic model of LIF is described as:


(1) $$\begin{array}{l} H\left[t\right]=V\left[t-1\right]+\frac{1}{\tau }\left(X\left[t\right]-\left(V\left[t-1\right]-V_{reset}\right)\right), \end{array}$$

(2) $$\begin{array}{l} S\left[t\right]=\Theta \left(H\left[t\right]-V_{th}\right), \end{array}$$

(3) $$\begin{array}{l} V\left[t\right]=H\left[t\right]\left(1-S\left[t\right]\right)+V_{reset}S\left[t\right], \end{array}$$

where τ is the membrane time constant, and *X*[*t*] is the input current at time step *t*. When the membrane potential *H*[*t*] exceeds the firing threshold *V*_*th*_, the spiking neuron triggers a spike *S*[*t*]. Θ(·) is the Heaviside step function which equals 1 for *v*≥0 and 0 otherwise. *V*[*t*] represents the membrane potential after the trigger event which equals *H*[*t*] if no spike is generated, and otherwise equals to *V*_*reset*_.

### 3.2 Wavelet transform

Wavelet transforms (WTs) are powerful signal-processing tools that enable the localization of signals in both time and frequency domains, which is particularly useful for analyzing non-stationary signals like EEG. The discrete wavelet transform (DWT), in particular, provides an efficient method for multi-resolution analysis by decomposing signals into sub-bands corresponding to different frequency scales. This decomposition enables the extraction of local features at various scales, making it well-suited for EEG signal processing. EEG signals are nonlinear and non-stationary, posing challenges for traditional analysis methods in capturing their time-varying and multiscale nature. Wavelet transforms, and specifically DWT, offer a significant advantage in feature extraction and time-frequency characterization of EEG signals. The DWT decomposes EEG data into frequency bands such as delta (0.5–4 Hz), theta (4–8 Hz), alpha (8–13 Hz), beta (13–30 Hz), and gamma (greater than 30 Hz). This decomposition allows us to extract meaningful features from the EEG data that correspond to various cognitive and emotional states.

For our application, we employ the Haar wavelet due to its simplicity and computational efficiency. Haar wavelets are among the earliest and simplest wavelet functions, characterized by a two-tap filter with minimal support, which results in fast computations. Compared to other common wavelets like Daubechies or Morlet, Haar wavelets are computationally less expensive, requiring only additions and binary shifts, which makes them well-suited for real-time, low-power applications such as SNN-based systems. Haar wavelets are particularly efficient in extracting local, low-frequency components (such as delta and theta waves) as well as high-frequency components (like beta and gamma waves), which are essential for distinguishing different cognitive states in EEG analysis. The efficiency and simplicity of Haar wavelets also make them ideal for handling the sparse, event-driven nature of SNNs.

### 3.3 Spiking self attention mechanism

The Transformer architecture, originally devised for natural language processing tasks (Vaswani et al., 2017), has subsequently permeated multiple subfields of artificial intelligence. At its core lies the self-attention mechanism, which facilitates selective information processing by focusing on relevant contextual elements. Spikformer (Zhou et al., 2022) pioneered the integration of self-attention into SNNs through their Spiking Self-Attention (SSA) framework and spikformer architecture. This approach innovatively employs sparse spiking representations for the query (*Q*), key (*K*), and value (*V*) matrices:


(4) $$\begin{array}{l} Q=SN\left(\text{BN}\left(XW_{Q}\right)\right), \end{array}$$

(5) $$\begin{array}{l} K=SN\left(\text{BN}\left(XW_{K}\right)\right), \end{array}$$

(6) $$\begin{array}{l} V=SN\left(\text{BN}\left(XW_{V}\right)\right), \end{array}$$

here, *Q*, *K*, and *V* form tensors of dimension ℝ^*T*×*C*×*H*×*W*^, with BN(·) representing batch normalization and SN(·) denoting the spiking neuron layer that maintains the attention mechanism's spiking nature. The similarity computation between spiking *Q* and *K* matrices proceeds via dot-product:


(7) $$\begin{array}{l} \text{Score}=\text{Sim}\left(Q,K\right)=QK^{⊤}. \end{array}$$

The attention output is subsequently calculated as a scaled weighted sum of *V*, transformed through spiking neuron activation, and further processed through linear transformation and batch normalization before final spiking neuron conversion to produce the output *Z*:


(8) $$\begin{array}{l} \text{Attn}=SN\left(s\cdot \text{Score}\cdot V\right),\;Z=SN\left(\text{BN}\left(\text{Linear}\left(\text{Attn}\right)\right)\right). \end{array}$$

## 4 Methods

In this section, we introduce our approach for EEG-based emotion recognition and auditory attention decoding. First, we define the problem formulation in Section 4.1. Then, we describe the overall data processing workflow in Section 4.2. Finally, we present the proposed Spiking Wavelet Transformer (SpikeWavformer) architecture which integrates wavelet transform and self spiking attention mechanisms in Section 4.4.

### 4.1 Problem analysis

Given an EEG dataset $D_{\text{eeg}}$, it can be represented as:


(9) $$\begin{array}{l} D_{\text{eeg}}={\left\{\left({\text{x}}_{i}^{\text{eeg}},y_{i}\right)\right\}}_{i=1}^{N}, \end{array}$$

where ${\text{x}}_{i}^{\text{eeg}}\in X_{\text{eeg}}$ denotes the raw EEG input signal for the *i*-th sample, and $y_{i}\in Y$ represents its corresponding label (emotion category or auditory attention state). Our objective is to learn a spiking neural network model *F*_θ_ with parameters θ to predict the class label from the EEG input. The model is optimized by minimizing the expected risk based on the cross-entropy loss *L*_CE_:


(10) $$\begin{array}{l} \arg\underset{\theta }{\min}\underset{\left({\text{x}}^{\text{eeg}},y\right)\sim D_{\text{eeg}}}{E}\left[L_{\text{CE}}\left(F_{\theta }\left({\text{x}}^{\text{eeg}}\right),y\right)\right]. \end{array}$$

In this study, we present a novel spiking transformer model, denoted as *F*_θ_, to learn discriminative spatio-temporal representations directly from raw EEG signals for the joint tasks of emotion recognition and auditory attention decoding. To achieve this, we introduce a novel Spiking Wavelet Self-Attention (SWSA) mechanism within a spiking transformer framework. While conventional Spiking Self-Attention (SSA) enables efficient event-driven computation, it is limited in its ability to capture the multi-scale frequency dynamics intrinsic to non-stationary EEG signals. The proposed SWSA overcomes this limitation by integrating Haar wavelet transforms for joint time—frequency analysis, which offer a minimal filter length and computational simplicity, making them highly efficient for real-time processing. Compared to other wavelet bases, such as Daubechies and Morlet, Haar's shorter filters and multiply-free operations align well with the event-driven, low-power nature of spiking neurons. This integration allows the model to focus on neurophysiologically relevant rhythms (e.g., alpha and beta bands) critical for emotional and attentional processes, while maintaining energy-efficient computation. Finally, a cross-entropy loss function is employed to enable effective gradient-based optimization for learning highly discriminative features across both tasks.

### 4.2 Workflow

**Figure 2:**
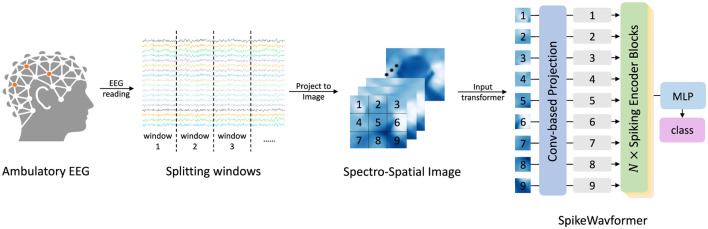
Workflow of the proposed method for EEG-based tasks. First, raw EEG data are preprocessed and segmented via sliding windows. Second, the α-band cortical activity is visualized as 2D topological maps. Finally, the data are tokenized into fixed-length sequences with multiple spiking encoder blocks performing feature extraction and an MLP head outputting the predicted category.

The overall workflow of the proposed method is depicted in Figure 2. Raw EEG signals are first preprocessed and segmented into overlapping windows via a sliding window strategy to preserve temporal continuity. To more effectively capture the spatial characteristics of EEG activity, α-band cortical signals are extracted and projected onto 2D topographic maps, thereby maintaining brain-region dependencies. These maps are subsequently divided into patches and tokenized into fixed-length sequences, which serve as the input to a stack of *N* spiking encoder blocks. Finally, the resulting features are fed into an MLP classification head to predict the corresponding emotional or attentional state. In summary, this high-level pipeline constitutes the basis of the proposed model architecture, which is elaborated in the following section.

### 4.3 SpikeWavformer

**Figure 3:**
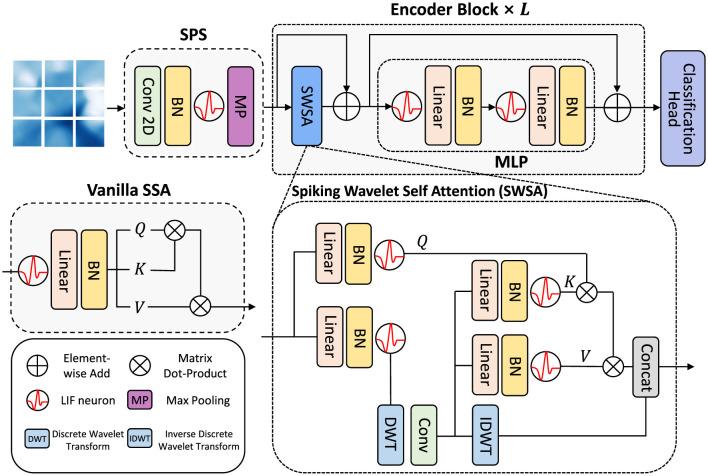
The overall architecture of our proposed Spiking Wavelet Transformer (SpikeWavformer) for EEG-based tasks, which consists of a spiking patch splitting module, *L*× spiking wavelet encoder blocks, and a linear classification head.

Building on the workflow described above, we design SpikeWavformer—an end-to-end spiking transformer architecture that combines wavelet-based multiscale analysis with spiking attention to enhance EEG feature representation. As shown in Figure 3, The SpikeWavformer can be written as follows:


(11) $$\begin{array}{l} X=\text{SPS}\left(X\right),\\ X_{l}^{\prime }=\text{SWSA}\left(X_{l-1}\right)+X_{l-1},\;l=1,\ldots ,L\\ X_{l}=\text{MLP}\left(X_{l}^{\prime }\right)+X_{l}^{\prime },\;l=1,\ldots ,L\\ Y=\text{CH}\left(\text{GAP}\left(X_{L}\right)\right). \end{array}$$

Given the EEG input *X*, SpikeWavformer first visualizes the spatial focus position via the topographic distribution of oscillatory cortical activities in the α band and converts it into a 2D image. Subsequently, the SPS module partitions the input into patches and progressively extracts features, optionally incorporating wavelet transformation to enhance multiscale feature representation. Then, *L*× spiking wavelet encoder blocks with spiking wave attention mechanism are employed to encode the features. Finally, the features obtained from extraction and encoding are compressed into a fixed-dimension vector via global average pooling (GAP) and fed into a fully connected layer classification head (CH) to produce classification results.

### 4.4 Spiking wavelet encoder block

As an essential neurophysiological signal, EEG plays a pivotal role in research areas such as affective computing and auditory attention decoding. Nevertheless, its multi-channel structure, low signal-to-noise ratio (SNR), pronounced temporal non-stationarity, and intricate time–frequency characteristics present substantial challenges for existing analysis techniques. Conventional CNNs are limited in capturing long-range temporal dependencies inherent in EEG data. In contrast, vanilla Transformers possess strong long-range modeling capability but incur prohibitive computational costs when processing long-sequence EEG signals. Furthermore, many existing approaches employ irreversible downsampling during multi-scale feature extraction, resulting in the loss of critical frequency-domain information. This drawback is particularly detrimental to neural decoding tasks that rely on specific frequency bands.

To address these issues, we propose a Spiking Wavelet Self-Attention (SWSA) mechanism for EEG signal processing. It combines the biological plausibility of SNNs with the flexible time-frequency analysis of wavelet transforms, offering an efficient, biologically inspired solution for EEG-based emotion recognition and auditory attention decoding. Specifically, given multi-channel EEG inputs *X*∈ℝ^*T*×*B*×*C*×*H*×*W*^, where *T* denotes time steps, *B* batch size, *C* EEG channels, and *H*×*W* spatial-topological 2D arrangement. The frequency-domain features of EEG signals are crucial for neuro-decoding. Different frequency bands correspond to different cognitive states: δ with deep sleep, θ with memory encoding, α with relaxation, β with attention and cognitive activities, and γ with perception and higher-order functions. We adopt the Haar wavelet for its minimal filter length and computational simplicity, which enable fast, low-power multiscale decomposition and align well with the event-driven, resource-constrained nature of SNN-based BCI systems. Specifically, the Haar wavelet is used for multiscale decomposition and perform DWT on EEG features at each time step *t*:


(12) $$\begin{array}{l} \left[X_{LL}^{\left(t\right)},X_{LH}^{\left(t\right)},X_{HL}^{\left(t\right)},X_{HH}^{\left(t\right)}\right]=\mathrm{DWT}\left(X^{\left(t\right)}\right), \end{array}$$

here, $X_{LL}^{\left(t\right)}$ captures low-frequency components (like δ, θ), while high-frequency sub-bands $X_{LH}^{\left(t\right)}$, $X_{HL}^{\left(t\right)}$, $X_{HH}^{\left(t\right)}$ retain high-frequency information (β, γ). Then, spatial local convolution enhances frequency-band interactions:


(13) $$\begin{array}{l} X_{filt}^{\left(t\right)}=\text{LIF}\left(\text{BN}\left(\text{Conv}\left(\text{Concat}\left(\left[X_{LL}^{\left(t\right)},X_{LH}^{\left(t\right)},X_{HL}^{\left(t\right)}\right]\right)\right)\right)\right), \end{array}$$

here, BN is batch normalization, LIF a spiking neuron layer. IDWT reconstructs spatial-domain features:


(14) $$\begin{array}{l} X_{recon}^{\left(t\right)}=\mathrm{IDWT}\left(X_{filt}^{\left(t\right)}\right). \end{array}$$

Our encoder, inspired by vanilla encoder (Vaswani et al., 2017), first calculates block-input spikes for self-attention. Three matrices $W_{q}\in {\mathbb{R}}^{d\times d_{q}}$, $W_{k}\in {\mathbb{R}}^{d\times d_{k}}$, $W_{v}\in {\mathbb{R}}^{d\times d_{v}}$ map tokens to vectors. Spiking neurons convert vectors to spiking sequences *Q*, *K*, *V*:


(15) $$\begin{array}{l} Q=\text{LIF}\left(\text{BN}\left(XW_{q}\right)\right),K=\text{LIF}\left(\text{BN}\left(XW_{k}\right)\right),V=\text{LIF}\left(\text{BN}\left(XW_{v}\right)\right). \end{array}$$

Next, we compute *Q*-*K* similarity. Following Zhou et al. (2022), a scaling factor *s* controls matrix-multiplication magnitude without affecting attention properties:


(16) $$\begin{array}{l} X_{\text{attn}}=\text{LIF}\left(QK^{⊤}V*s\right), \end{array}$$

(17) $$\begin{array}{l} X_{\text{attn}}^{\prime }=\text{LIF}\left(\text{BN}\left(\text{Linear}\left(X_{\text{attn}}\right)\right)\right). \end{array}$$

To integrate wavelet and attention features effectively, we use channel-wise concatenation:


(18) $$\begin{array}{l} X_{\text{combined}}=\text{Concat}\left(X_{\text{attn}}^{\prime },X_{\text{recon}}^{\left(t\right)}\right), \end{array}$$

(19) $$\begin{array}{l} \text{SWSA}\left(X\right)=\text{LIF}\left(\text{BN}\left(X_{\text{combined}}\right)\right). \end{array}$$

By integrating wavelet decomposition with spiking mechanisms, SpikeWavformer enables efficient processing of long-sequence EEG data while facilitating the analysis of cross-frequency neural dynamics, thereby providing richer feature representations for complex neuro decoding tasks. Specifically, we analyze the advantages of integrating wavelet transform into SNNs from the perspectives of convergence and convergence speed. First, We define the EEG signal space as *X* = {*x*∈ℝ^*T*×*C*×*H*×*W*^}, where *T* represents time steps. *C* denotes channels, and *H*×*W* represents spatial dimensions. The discrete wavelet transform operator is defined as:


(20) $$\begin{array}{l} W:X\to Y, \end{array}$$

where *Y* = {(*X*_*LL*_, *X*_*LH*_, *X*_*HL*_, *X*_*HH*_)} represents the wavelet coefficient space.

The SWSA mechanism can be formalized as a composite operator:


(21) $$\begin{array}{l} \text{SWSA}\left(X\right)=F\left(\text{Attn}\left(W\left(X\right)\right)\right)⊕W^{-1}\left(W\left(X\right)\right), \end{array}$$

where *W* is the DWT operator, Attn is the spiking attention operator, *F* is the fusion operator, ⊕ denotes concatenation and *W*^−1^ is the inverse DWT (IDWT).

**Theorem 1 (Lipschtiz Continuity of SWSA):** The SWSA mechanism satisfies Lipschitz continuity (Hager, 1979; Gouk et al., 2021; Goldstein, 1977) with constant *L*_*SWSA*_, ensuring stable convergence during training.

**Proof:** First, we establish the Lipschitz properties of individual components: ***Haar Wavelet Transform Lipschitz Property:*** For the Haar wavelet transform *W*, we have: ||*W*(*x*_1_)−*W*(*x*_2_)||_2_ ≤ *L*_*W*_||*x*_1_−*x*_2_||_2_. Since Haar wavelets are orthonormal, *L*_*W*_ = 1. ***Spiking Attention Lipschitz Property:*** For the spiking attention mechanism with LIF neuron, let ϕ(μ) = Θ(μ−*V*_*th*_) be the spike generation function. The membrane potential dynamics: *V*[*t*] = τ*V*[*t*−1]+*X*[*t*]−*v*_*reset*_*S*[*t*−1]. For bounded inputs, the LIF neuron satisfies: $\left|\phi \left(\mu_{1}\right)-\phi \left(\mu_{2}\right)|\leq \frac{1}{V_{th}}|\mu_{1}-\mu_{2}\right|$. Therefore, $L_{A}=\frac{1}{V_{th}}$. ***Combined Operator:*** The SWSA operator combines these components: ||SWSA(*x*_1_)−SWSA(*x*_2_)||_2_ ≤ *L*_*SWSA*_||*x*_1_−*x*_2_||_2_, where $L_{SWSA}=L_{W}\cdot L_{A}\cdot L_{F}=\frac{L_{F}}{V_{th}}$ with *L*_*F*_ being the Lipschitz constant of the fusion operation.

**Corollary 1:** Under the assumption that *L*_*SWSA*_ < 1, the SWSA operator is a contraction mapping, guaranteeing convergence to a unique fixed point.

**Theorem 2 (Accelerated convergence):** The SWSA mechanism achieves faster convergence compared to vanilla spiking self attention.

**Proof:** Consider the optimization landscape with loss function *L*(θ). The gradient update for SWSA parameters follows: θ_*t*+1_ = θ_*t*_−α∇_θ_*L*(θ_*t*_). The wavelet decomposition provides a natural regularization through frequency localization:


(22) $$\begin{array}{l} L_{SWSA}\left(\theta \right)=L_{data}\left(\theta \right)+\lambda \underset{j}{\sum }||W_{j}|{|}_{1}. \end{array}$$

This *L*_1_ regularization on wavelet coefficients promotes sparsity. The convergence rate is bounded by:


(23) $$\begin{array}{l} L\left(\theta_{r}\right)-L^{*}\leq \frac{1}{2\alpha T}||\theta_{0}-\theta^{*}|{|}^{2}+\frac{\alpha L}{2}\sigma^{2}, \end{array}$$

where the wavelet regularization reduces the effective variance σ^2^, leading to faster convergence.

## 5 Experiment

This section presents comprehensive experiments to evaluate the effectiveness and efficiency of the proposed SpikeWavformer model. First, we detail the experimental setup, including datasets, preprocessing, and implementation specifics. Second, comparative studies are conducted on the DEAP and KUL datasets, demonstrating superior performance over existing methods in both emotion recognition and auditory attention decoding tasks. Additionally, we provide an analysis of the model's energy efficiency, highlighting its advantages in low-power computing environments.

### 5.1 Experimental setup

#### 5.1.1 Datasets

**DEAP**. The DEAP dataset (Koelstra et al., 2011), widely used in emotion recognition research, examines emotional responses to multimedia stimuli by employing peripheral physiological data and EEG signals. It includes 32-channel EEG recordings and various physiological signals, such as skin temperature, blood volume pulse (BVP), respiratory rate, galvanic skin response (GSR), electrooculogram (EOG), and video clips of facial expressions. The facial expressions of the first 22 participants were also recorded. Each participant completed 40 trials, with each trial lasting 1 min and a 3-second baseline recorded before the start of each trial. After each trial, participants filled out a questionnaire to self-report their emotional state in terms of arousal, valence, dominance, and liking, with each dimension rated on a 9-point scale. EEG data were collected using a 32-channel device at a sampling rate of 512 Hz.

**KUL**. The KUL dataset (Das et al., 2019) comprises EEG data collected using the BioSemi ActivateTwo device. The experimental environment was electromagnetically shielded and soundproofed to minimize potential noise interference. Data were collected from 16 subjects with normal hearing, who were instructed to focus on a specific speaker amidst two speakers. The speakers narrated four Dutch stories. Each subject participated in 8 trials, each lasting 6 min. Auditory stimuli, filtered through HRTF, were presented to the subjects in two forms: from the left or right side, in a randomized manner.

#### 5.1.2 Implementation details

The EEG data from each channel was first re-referenced to the average response of all electrodes. Given that the analyzed EEG signals were collected at different sampling rates, they were all band-pass filtered between 1 and 32 Hz using a 6th-order Chebyshev Type II filter and down sampled to a 128 Hz sampling rate. The frequency range was chosen based on previous nonlinear AAD studies. Finally, the EEG data channels were normalized to ensure a mean of zero and unit variance for each trial. The study on the KUL dataset analyzed seven decision window sizes: 0.1, 0.2, 0.5, 1, 2, 5, and 10 seconds. Experiments were conducted using two NVIDIA RTX 4090 GPUs. The model was optimized using the Adam optimizer with an initial learning rate of 1 × 10^−4^ and trained for 200 epochs. For the SNN model parameters, LIF neurons were set with an initial membrane potential of 0, a spiking threshold of 0.5, and a simulation time step of 4. To facilitate effective backpropagation, a sigmoid function with parameter α = 4 was used as the surrogate gradient function, expressed as *sigmoid*(*x*) = 1/(1+*exp*(−α*x*)). The remaining setup of spiking transformer architecture follows spikformer (Zhou et al., 2022).

### 5.2 Comparative study

We conduct experiments on the DEAP and KUL datasets using proposed SpikeWavformer and compare the results with existing methods for emotion recognition and auditory attention decoding. As shown in Tables 1, 2, our method achieves state-of-the-art performance on all datasets. On the DEAP dataset for emotion recognition, the SpikeWavformer method reaches an Arousal accuracy of 76.51% (std: 5.48%) and a Valence accuracy of 77.10% (std: 5.68%). Existing methods like EEGNet (Lawhern et al., 2018) achieve 58.29% (std: 8.60%) for Arousal and 54.56% (std: 8.14%) for Valence. SCN (Schirrmeister et al., 2017) attains 61.19% (std: 10.28%) for Arousal and 59.42% (std: 8.30%) for Valence. DCN (Schirrmeister et al., 2017) gets 61.03% (std: 8.58%) for Arousal and 59.92% (std: 7.82%) for Valence. Tsception (Ding et al., 2022) achieves 61.57% (std: 11.04%) for Arousal and 59.14% (std: 7.60%) for Valence.

**Table 1:** Comparison of different methods on DEAP dataset.

**Dataset**	**Method**	**Arousal**		**Valence**	
		**Acc**.	**Std**	**Acc**.	**Std**
DEAP	EEGNet (Lawhern et al., 2018)	58.29%	8.60%	54.56%	8.14%
	SCN (Schirrmeister et al., 2017)	61.19%	10.28%	59.42%	8.30%
	DCN (Schirrmeister et al., 2017)	61.03%	8.58%	59.92%	7.82%
	Tsception (Ding et al., 2022)	61.57%	11.04%	59.14%	7.60%
	**SpikeWavformer**	**76.51%**	**5.48%**	**77.10%**	**5.68%**

The bold text refers to the method proposed in this paper.

**Table 2:** Performance comparison across different decision windows.

**Dataset**	**Model**	**Decision window (second)**						
		**0.1**	**0.2**	**0.5**	**1**	**2**	**5**	**10**
KUL	Linear (CCA) (De Cheveigné et al., 2018)	50.9	53.6	55.7	60.2	63.5	69.4	75.9
	Non-linear (CNN) (Cai et al., 2021)	74.3	78.2	80.6	84.1	85.7	86.9	87.9
	STAnet (Su et al., 2022)	80.8	84.3	87.2	90.1	91.4	92.6	93.9
	**SpikeWavformer**	**80.5**	**86.7**	**94.2**	**96.5**	**97.1**	**97.3**	**98.6**

The bold text refers to the method proposed in this paper.

We further compared the performance of the SpikeWavformer for different detection window sizes, ranging from 0.1 to 10 seconds, with the results presented in Table 2. On the KUL dataset, the SpikeWavformer achieved an average decoding accuracy of 96.5% across all subjects for a 1-second decision window, 97.1% for a 2-second decision window, 97.3% for a 5-second decision window, and 98.6% for a 10-second decision window. Generally, larger decision windows yielded better results, corroborating findings from previous studies (De Taillez et al., 2020; Ciccarelli et al., 2019; Vandecappelle et al., 2021). Notably, our proposed method is capable of decoding auditory spatial attention with a very short decision window of less than 1 second. For decision windows of 0.5 seconds and 0.2 seconds, the SpikeWavformer attained high accuracy rates of 94.2% and 86.7%, respectively. Although the accuracy for the 0.1-second decision window was lower than that of the 1-second decision window, SpikeWavformer maintained a high accuracy rate of 80.5%. In all comparisons with related work (De Cheveigné et al., 2018; Cai et al., 2021; Su et al., 2022), the SpikeWavformer demonstrated competitive performance.

### 5.3 Energy consumption comparison

In this section, we validate the energy efficiency of our proposed model over its ANN counterpart. Based on the energy calculation standard in neuromorphic computing (Sengupta et al., 2019), we use the method proposed by Wang et al. (2024) to compute the energy consumption ratio between our model and the equivalent ANN model:


(24) $$\begin{array}{l} {\text{Energy}}_{\text{rate}}=\frac{\text{AC}}{\text{MAC}}*\text{SpikingRate}*\text{TimeSteps}. \end{array}$$

In the equation, $\frac{\text{AC}}{\text{MAC}}$ denotes the energy consumption ratio of an accumulate (AC) operation in SNNs to a multiplication (MAC) in ANNs. Extensive studies confirm the theoretical value of $\frac{\text{AC}}{\text{MAC}}$ is $\frac{1}{7}$ (Horowitz, 2014). Here, SpikingRate is the average spiking rate, and TimeSteps the simulation time window. In our model, SpikingRate is 12.3%, and TimeSteps is set to 4. Based on Equation 24, our model achieves over 7× energy efficiency compared to its ANN counterpart.

### 5.4 Interpretability

**Figure 4:**
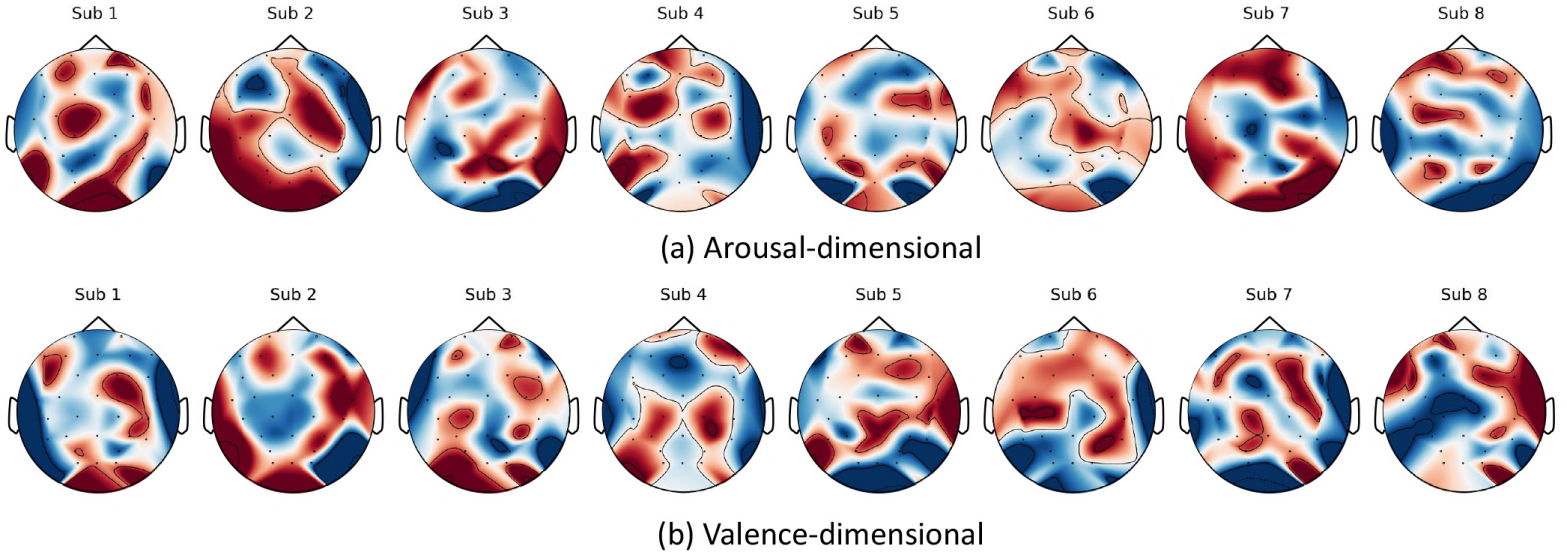
Visualization of saliency maps from DEAP dataset (Sub 1–8): **(a)** Arousal-dimensional saliency maps and **(b)** valence-dimensional saliency maps.

**Figure 5:**
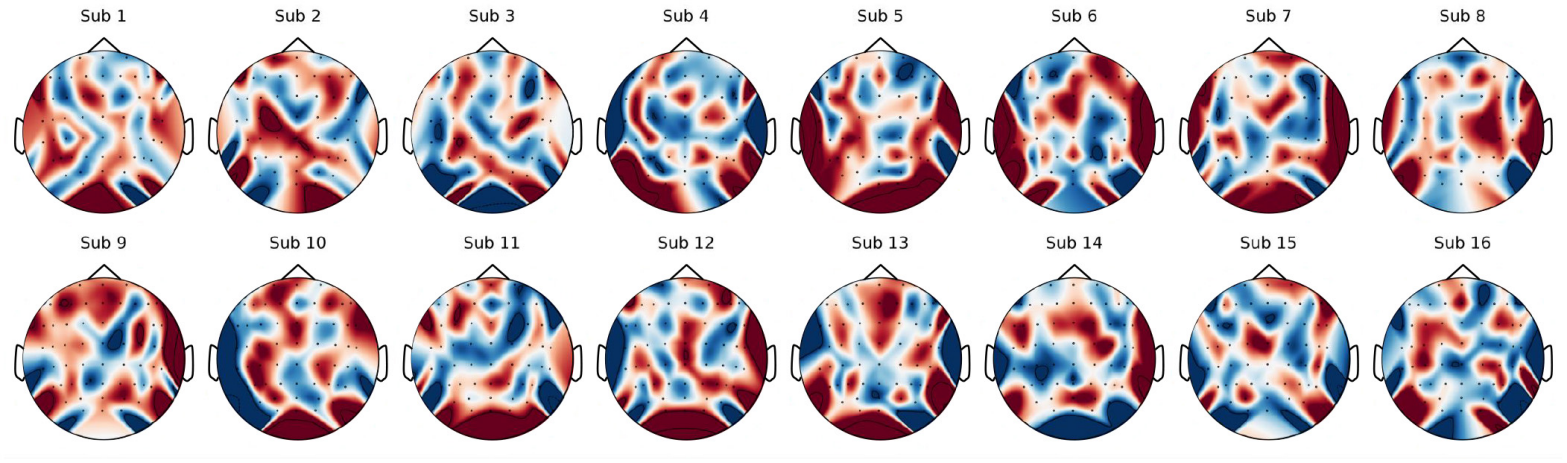
Visualization of Arousal-dimensional saliency maps from KUL dataset (Sub 1–16).

In this section, saliency maps (Simonyan et al., 2013) are employed to visualize the areas of the data that contain the most information and contribute to classification performance. The saliency map is one of the most widely used tools for illustrating which regions of the input data hold classification-relevant information. To enhance the visualization of the saliency maps, the original maps were averaged along the time dimension to capture the topology of the EEG channels. Additionally, the normalized saliency maps were averaged across different samples for each subject to produce generalized average saliency maps. The average saliency maps for the DEAP dataset and the KUL dataset are presented in Figures 4, 5, respectively.

**DEAP**. For arousal, as illustrated in Figure 4a, the temporal and frontal regions of the brain contain a wealth of information. This indicates that these regions are more involved in processing emotions, aligning with findings from previous studies (Gao et al., 2021; Huang et al., 2012; Mickley Steinmetz and Kensinger, 2009). Emotional arousal is predominantly represented in the temporal and frontal lobes. The asymmetry between the frontal and temporal lobes is closely associated with emotion recognition within the arousal dimension. In terms of valence, Figure 4b shows that the parietal and temporal lobes are also rich in information. This observation is consistent with earlier research (Huang et al., 2012), suggesting that the network effectively learns from these relevant regions.

**KUL**. It is expected that the areas of neural activity contributing to speech processing will exhibit greater significance. As illustrated in Figure 5, the average saliency map of the KUL dataset reveals that the frontal and temporal regions contain more substantial information. These findings align with previous research indicating that activation is prominently observed in the frontal and temporal cortices (Ciccarelli et al., 2019; Geirnaert et al., 2020; Vandecappelle et al., 2021).

## 6 Conclusion

This paper presents SpikeWavformer, an end-to-end deep learning SNN model that integrates the wavelet transform with spiking transformer architecture. The model combines the global–local feature extraction capability of the wavelet transform with the low-power, event-driven computation of spiking neurons, enabling dynamic modeling and efficient processing of EEG signals. This integration supports effective time–frequency decomposition, automatic feature extraction, and classification, thereby improving generalization across diverse scenarios. Experiments on two publicly available datasets demonstrate that SpikeWavformer consistently outperforms established methods. The experimental results validate its effectiveness in both emotion recognition and auditory attention decoding tasks, highlighting its potential for deployment in resource-constrained brain–computer interface applications. Future deployment of SpikeWavformer on neuromorphic hardware platforms presents both promising opportunities and technical challenges. The energy-efficient characteristics of the approach make it particularly well-suited for implementation on neuromorphic chips, potentially enabling low-power BCI applications in portable devices. However, contemporary neuromorphic architectures are primarily optimized for convolution-based SNNs, necessitating further hardware–software co-design efforts to fully realize the benefits of Transformer-based spiking architectures. Overall, this study advances the development of energy-efficient, high-performance brain–computer interfaces suitable for resource-constrained practical deployment.

## Data Availability

The datasets used in this study are publicly available. The dataset DEAP for this study can be found at https://www.eecs.qmul.ac.uk/mmv/datasets/deap/. The dataset KUL for this study can be found at https://zenodo.org/records/4004271.
